# Supplementary material for: CAMUS: scalable phylogenetic network estimation
Source: Bioinformatics. 2026 Jul 7;42(Suppl 1):btag245. doi: 10.1093/bioinformatics/btag245 (PMC13340232; doi:10.1093/bioinformatics/btag245)
Supplement: btag245_Supplementary_Data [file btag245_supplementary_data.pdf]

Supplementary Materials for "CAMUS: Scalable Phylogenetic Network Estimation"

James Willson and Tandy Warnow

Contents

1 Data simulation
1.1 Model Networks . . . .
1.2 Gene Trees . . . .

2 Network estimation
2.1 CAMUS . . . .
2.2 SNaQ . . . .
2.3 PhyloNet-MPL . . . .

3 Additional results on synthetic datasets

4 Avian phylogenomics dataset analysis
4.1 Quartets not satisfied for different analyses of the Avian dataset . . . .
4.2 CAMUS analyses of the Avian dataset . . . .
4.3 PhyloNet-MPL(FT) analyses of the Avian dataset . . . .

5 References

List of Figures

S1 Distribution of the number of reticulations for our six model conditions. . . . .
S2 Impact of quartet filtering . . . .
S3 Lineplots showing number of quartets not satisfied in CAMUS analyses using ASTRAL trees on the avian dataset . . . .
S4 CAMUS network with one reticulation computed on the Avian constraint tree with $t = 0.5$ . . . .
S5 CAMUS network with one reticulation computed on the Avian constraint tree with $t = 0.8$ . . . .
S6 CAMUS network with two reticulations computed on the Avian constraint tree with $t = 0.5$ . . . .
S7 CAMUS network with two reticulations computed on the Avian constraint tree with $t = 0.8$ . . . .
S8 PhyloNet-MPL(FT) network with one reticulation computed on the Avian constraint tree . . . .
S9 PhyloNet-MPL(FT) network with two reticulations computed on the Avian constraint tree . . . .

List of Tables

S1 INDELible Parameters . . . .
S2 Gene tree estimation error (GTEE) for our six model conditions. . . . .
S3 Taxonomic mapping of scientific names to common names for the Avian trees. . . .

1 Data simulation

The following pipeline is used for data simulation:

- Generate model phylogenetic networks using SiPhyNetworks (v1.1.0) [1].
- Outgroup taxa are then added to these networks.
- True gene trees are then generated from these networks using PhyloCoalSimulations (v1.0.0) [2].
- Sequences are generated from these gene trees using INDELible (v1.03) [3].
- FastTree2 (v2.1.11) [4] or IQTree3 (v3.0.1) [5] is then used to generate estimated gene trees.

We simulated 50 replicates but only used 20 in the experiments.

1.1 Model Networks

Model phylogenetic networks are generated from n taxa, where $n \in \{15, 25, 50, 100, 150, 200\}$ (plus one outgroup). SiPhyNetwork (v1.1.0) has the following additional parameters: speciation rate λ, hybridization rate μ, and extinction rate ν, as well as a vector `hyperprobs` controlling the probability that lineages are generative, degenerative, or neutral. A function to determine inheritance proportions is also taken as input. 50 replicates are generated for all model conditions.

$\lambda = 1$ for all model conditions; `hyperprobs` $= \langle 0.5, 0.25, 0.25\rangle$ where the values are lineage generative, lineage degenerative, and lineage neutral, respectively. Inheritance proportions are drawn from a Beta(10,10) distribution.

In addition, the following filtering criteria were enforced: each network must be level-1, must have at least one reticulation, and must have exactly n taxa.

Both ν and μ were adjusted (primarily to make finding a set of model networks computationally feasible as both of these parameters affect the likelihood of a network being level-1).

- $n = 25$, $\nu = 0.2$, $\mu = 0.025$
- $n = 50$, $\nu = 0.2$, $\mu = 0.0025$
- $n = 100$, $\nu = 0.05$, $\mu = 0.0025$
- $n = 150$, $\nu = 0.005$, $\mu = 0.0025$
- $n = 200$, $\nu = 0.005$, $\mu = 0.001$

This process resulted in level-1 networks with the number of reticulations distributed as shown in Figure S1.

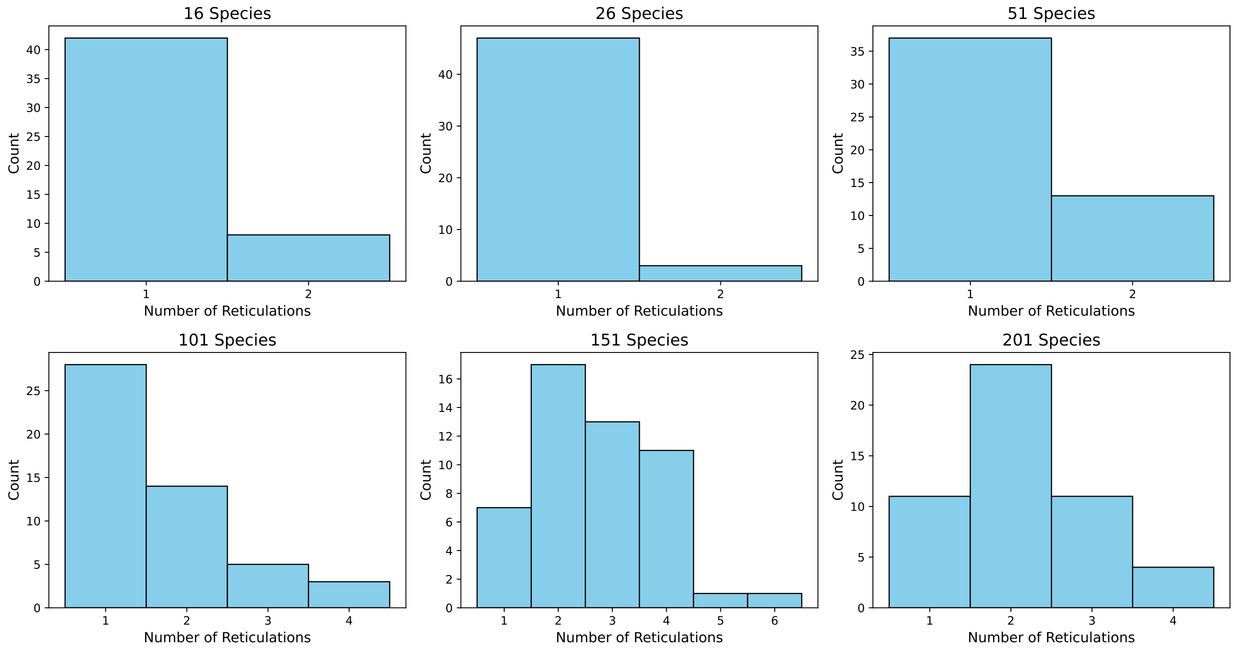

Figure S1: Distribution of the number of reticulations for our six model conditions.

Next, an outgroup taxa was added: a single vertex was created as the root, then the outgroup leaf was added with an edge length $[0.9, 1.0]$ (uniform distribution); finally, the previously generated network was attached to the other side of the root on the other side of a branch with length $[0.0, 0.1]$.

To generate the networks, we used an R script, `generate-networks.R` at `https://gist.github.com/jsdoublel/d15fb3ea6520bf13e2a87da7bcc0c42c` and then add outgroups to these networks with a python script `add-outgroup.py` at `https://gist.github.com/jsdoublel/de9ab383e0734d53222e289d0a737870`, using the following commands:

```
Rscript generate-networks.R $ntaxa 50 $nu $mu
find "n$ntaxa" -name "true_net.nwk" -exec python add-outgroup.py {} \;
```

1.2 Gene Trees

True gene trees were simulated from the model networks using PhyloCoalSimulations (v1.0.0) using default settings (i.e., single individual per species). Then sequences are generated with INDELible (v1.03). All sequences are generated a with length of 500 bp. This resulted in trees with $\approx 21\%$ gene tree estimation error.

To run INDELible we used the same process and scripts as in [6] (see that paper's Supplementary Materials for more details). We have included a table with the parameters used below (Table S1).

Table S1: INDELible Parameters

Parameters	Values
Base Frequencies	Dirichlet(T=113.48869,C=69.02545,A=78.66144,G=9983793)
Transition Rate	Dirichlet(CT=12.776722,AT=20.869581,GT=5.647810, AC=9.863668,GC=30.679899,AG=3.199725)
Gamma rate variation	Log-normal(μ-0.470703916, σ=0.348667224)

We estimated maximum likelihood trees under the Generalized Time Reversible (GTR) model [7] on these alignments, using either FastTree (v2.1.11) or IQTree3 (v3.0.1). We used FastTree (v2.1.11) with the following command:

```
fasttree -nt -gtr $input > $output
```

We used IQTree3 (v3.0.1) with the following command:

```
iqtree3 -T 32 -s $input -m GTR+G -bb 1000
```

The resulting amounts of gene tree estimation error for our different model conditions are shown in Tabel S2.

Table S2: Gene tree estimation error (GTEE) for our six model conditions.

Num. Species	Method	GTEE
16	IQTree3	19.6%
26	IQTree3	19.5%
51	FastTree2	21.0%
101	FastTree2	21.1%
151	FastTree2	20.5%
201	FastTree2	20.7%

2 Network estimation

The following command was used to run ASTRAL-IV [8] (v1.23.4.6) for the starting/constraint networks.

```
astral4 -t 32 -o $output $input 2> $log
```

Trees are then rerooted on the outgroup using the python script `root-outgroup.py` as so.

```
python root-outgroup.py $tree
```

root-outgroup.py uses TreeSwift [9] as a dependancy.

2.1 CAMUS

CAMUS (v1.0.0) was run with the following command

```
camus -n 32 -q 2 -t $threshold -o $output $const_tree $gene_trees
```

Older versions of CAMUS required `-q 2` to enable quartet filtering. This is now turned on by default and thus is unnecessary.

2.2 SNaQ

For SNaQ (v1.1) [10], we used `countquartetsintrees(·)` from PhyloNetworks [11] to calculate the quartet concordance factors, then we pass these concordance factors directly to SNaQ. We use the script `run_snaq.jl` to achieve this. We call this script with the command

```
julia --threads=auto run_snaq.jl $gene_trees  \
    $start_tree $output 1 32
```

2.3 PhyloNet-MPL

For PhyloNet-MPL (v3.8.2) [12, 13] we used a Python wrapper script to create the nexus files: `run_phylonet.py`. Then, to run PhyloNet-MPL we used the command

```
python run_phylonet.py \
    -p PhyloNetv3_8_2.jar \
    -g $gene_trees \
    -s $start_tree \
    -r 1 \
    -n 32 > $log 2>&1
```

And for PhyloNet-MPL (FT) we add the `-f` argument to the script.

3 Additional results on synthetic datasets

We provide additional results here that were omitted from the main paper due to space constraints.

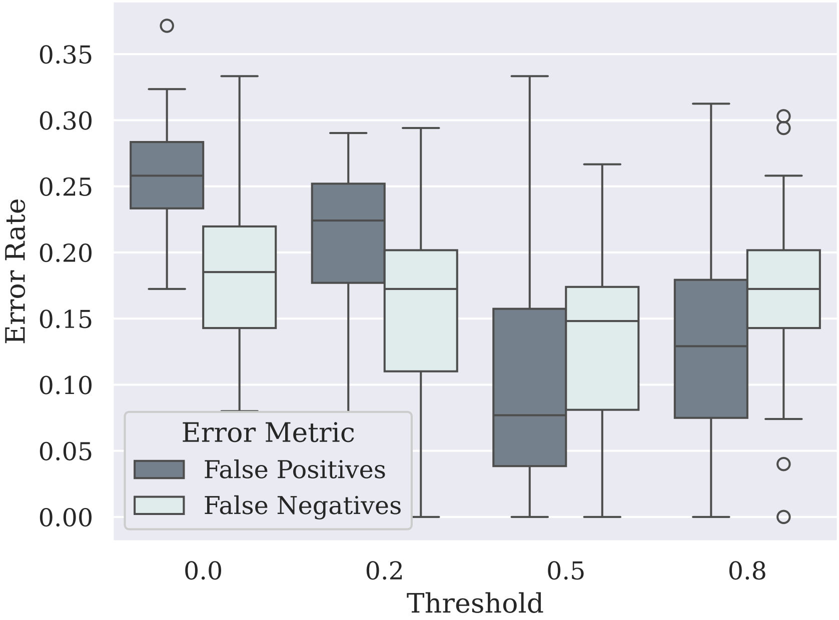

Figure S2: **Impact of quartet filtering threshold on phylogenetic network error rate computed by CAMUS.** Results are shown on the algorithm design datasets with 26-taxon FastTree gene trees where CAMUS adds one edge to produce 1 reticulation; we report the cluster metric error rate. Setting the threshold $t = 0.5$ produces the lowest error.

4 Avian phylogenomics dataset analysis

We examine the Avian dataset from [14] using both CAMUS and PhyloNet-MPL (FT); PhyloNet-MPL without a fixed starting tree was excluded, as it could not complete within 24 hours. We computed an ASTRAL tree for this experiment from the all the existing gene trees, taken from the original study [15]. The species names are given here using scientific names; the mapping to the common name is shown in Table S3.

Both CAMUS and PhyloNet-MPL(FT) use the gene trees to select how to add edges to the rooted constraint tree. We elected to collapse low support edges in the gene trees, based on standard treatment in phylogenomics; thus both methods received these modified gene trees.

Results shown in Figure S3 suggest that either one or two reticulations fit best. Therefore, we focus our analysis on CAMUS and PhyloNet-MPL(FT) for at most two reticulations, each using the ASTRAL tree (rooted at the outgroup), and each using the gene trees with low support (less than 75% bootstrap support) collapsed. For CAMUS, we elected to use $t = 0.5$ based on our prior experiments on simulated data; however, the gene trees in this study have very low average branch support (around 25%), suggesting potentially that setting $t = 0.8$ may be more appropriate. Hence, for CAMUS, we show results for two settings for t (0.5 and 0.8).

Commands and software versions used are identical to those provided earlier in this document. The dataset, including all gene trees, can be found at [15].

Table S3: Taxonomic mapping of scientific names to common names for the Avian trees.

**Scientific Name**	**Common Name**
Acanthisitta chloris	Rifleman
Anas platyrhynchos	Pekin Duck
Antrostomus carolinensis	Chuck-Will's-Widow
Apaloderma vittatum	Bar-Tailed Trogon
Aptenodytes forsteri	Emperor Penguin
Balearica regulorum	Grey-Crowned Crane
Buceros rhinoceros	Rhinoceros Hornbill
Calypte anna	Anna's Hummingbird
Cariama cristata	Red-Legged Seriema
Cathartes aura	Turkey Vulture
Chaetura pelagica	Chimney Swift
Charadrius vociferus	Killdeer
Chlamydotis macqueenii	MacQueen's Bustard
Columba livia	Pigeon

Continued on next page

Table S3 continued from previous page

**Scientific Name**	**Common Name**
Colius striatus	Speckled Mousebird
Corvus brachyrhynchos	American Crow
Cuculus canorus	Common Cuckoo
Egretta garzetta	Little Egret
Eurypyga helias	Sunbittern
Falco peregrinus	Peregrine Falcon
Fulmarus glacialis	Northern Fulmar
Gallus gallus	Chicken
Gavia stellata	Red-Throated Loon
Geospiza fortis	Medium Ground-Finch
Haliaeetus albicilla	White-Tailed Eagle
Haliaeetus leucocephalus	Bald Eagle
Leptosomus discolor	Cuckoo-Roller
Manacus vitellinus	Golden-Collared Manakin
Meleagris gallopavo	Turkey
Melopsittacus undulatus	Budgerigar
Merops nubicus	Carmine Bee-Eater
Mesitornis unicolor	Brown Mesite
Nestor notabilis	Kea
Nipponia nippon	Crested Ibis
Ophisthocomus hoazin	Hoatzin
Pelecanus crispus	Dalmatian Pelican
Phalacrocorax carbo	Great Cormorant
Phaethon lepturus	White-Tailed Tropicbird
Phoenicopterus ruber	American Flamingo
Picoides pubescens	Downy Woodpecker
Podiceps cristatus	Great-Crested Grebe
Pterocles gutturalis	Yellow-Throated Sandgrouse
Pygoscelis adeliae	Adelie Penguin
Struthio camelus	Ostrich
Taeniopygia guttata	Zebra Finch
Tauraco erythrolophus	Red-Crested Turaco
Tinamou guttatus	White-Throated Tinamou
Tyto alba	Barn Owl

4.1 Quartets not satisfied for different analyses of the Avian dataset

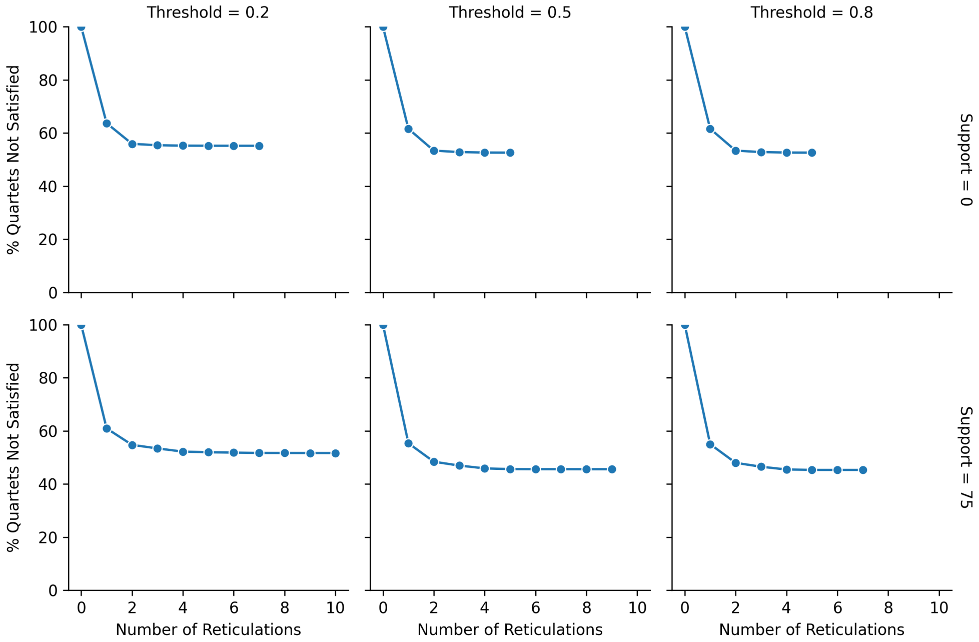

Figure S3: Lineplot showing the number of quartets not satisfied by CAMUS' outputted network for differing numbers of reticulations given an **ASTRAL constraint tree**. "Threshold" is the value of the quartet filter threshold t and "Support" is the cutoff where branches in the gene trees with support below that value were collapsed.

4.2 CAMUS analyses of the Avian dataset

In this section, we show results when we use CAMUS on species trees computed using ASTRAL, and the quartet set (Q) is based on gene trees where edges with bootstrap support less than 75% have been collapsed. Here we vary the threshold t and allow either 1 or 2 reticulations.

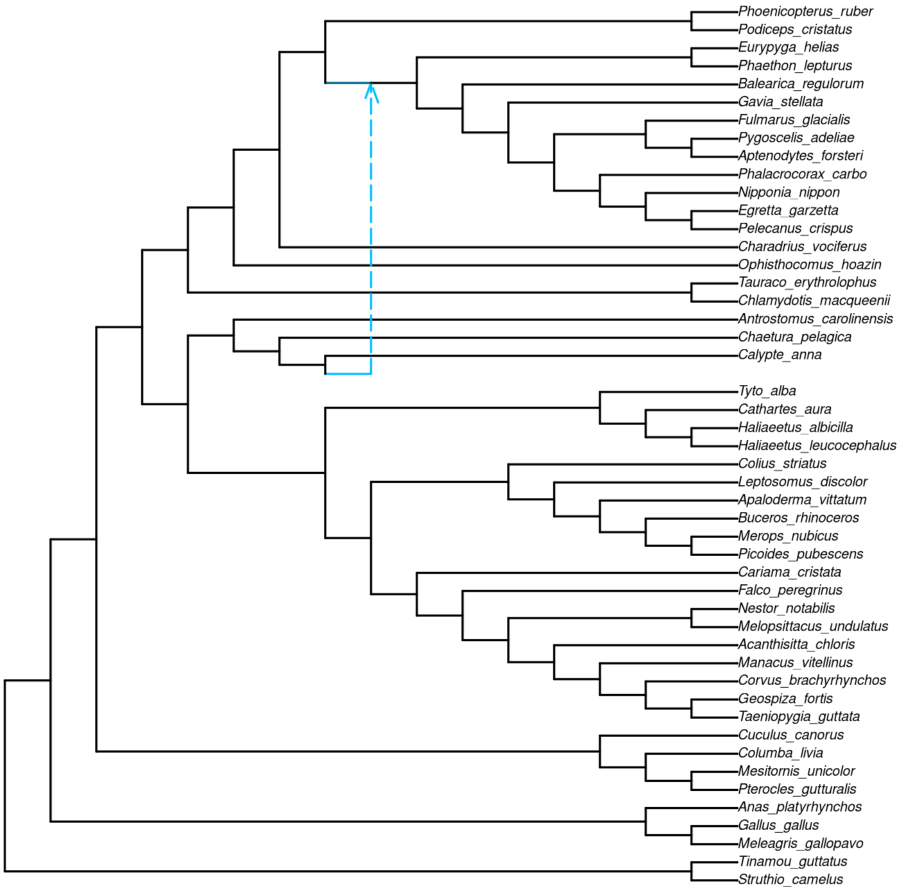

Figure S4: Network with one reticulation returned by CAMUS on the Avian dataset with ASTRAL constraint tree; $t = 0.5$; edges in gene trees with support less than 75% collapsed.

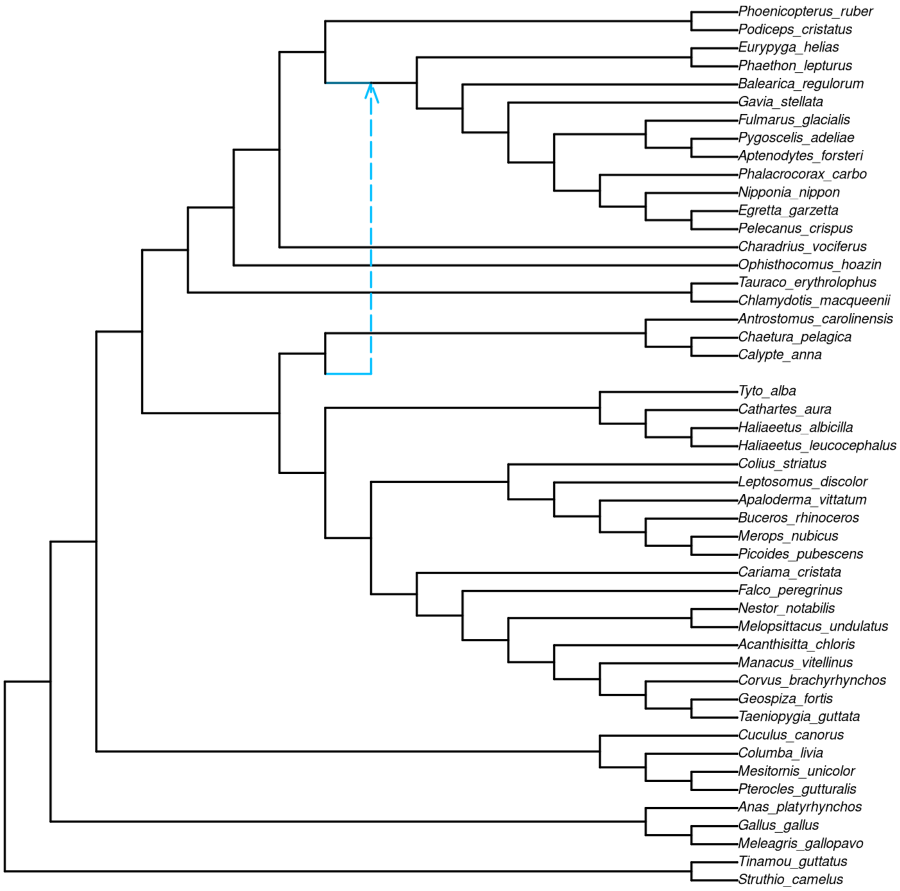

Figure S5: Network with one reticulation returned by CAMUS with ASTRAL constraint tree; $t = 0.8$; edges in gene trees with support less than 75% collapsed.

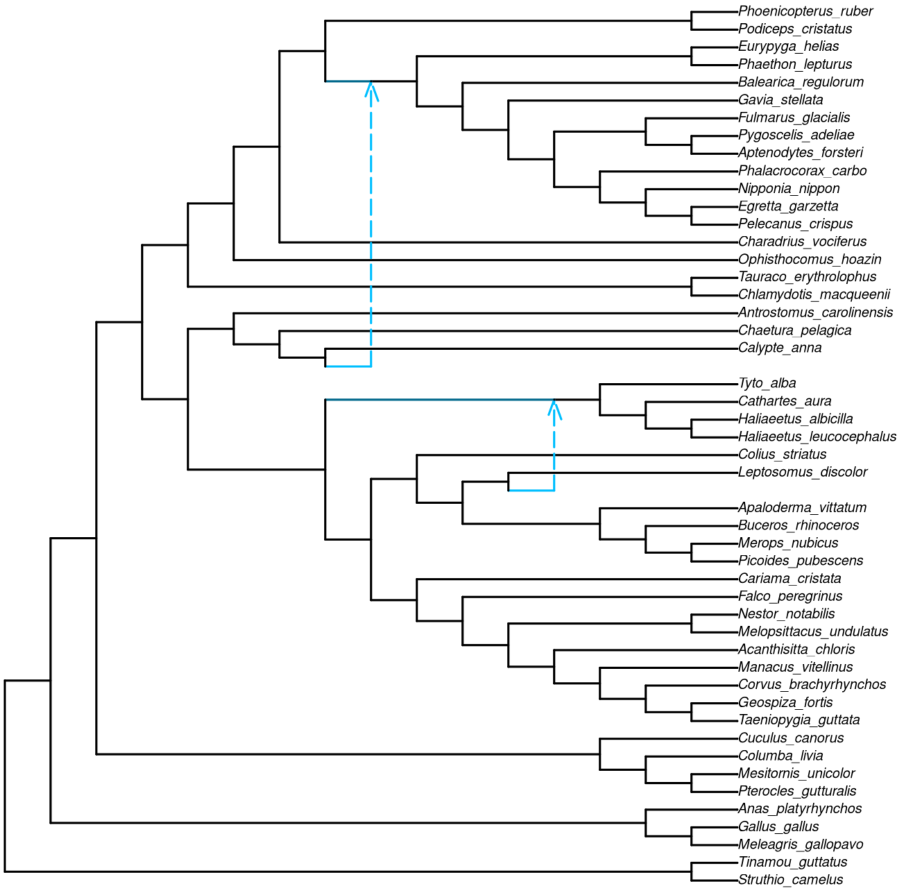

Figure S6: Network with two reticulations returned by CAMUS with ASTRAL constraint tree; $t = 0.5$; edges in gene trees with support less than 75% collapsed.

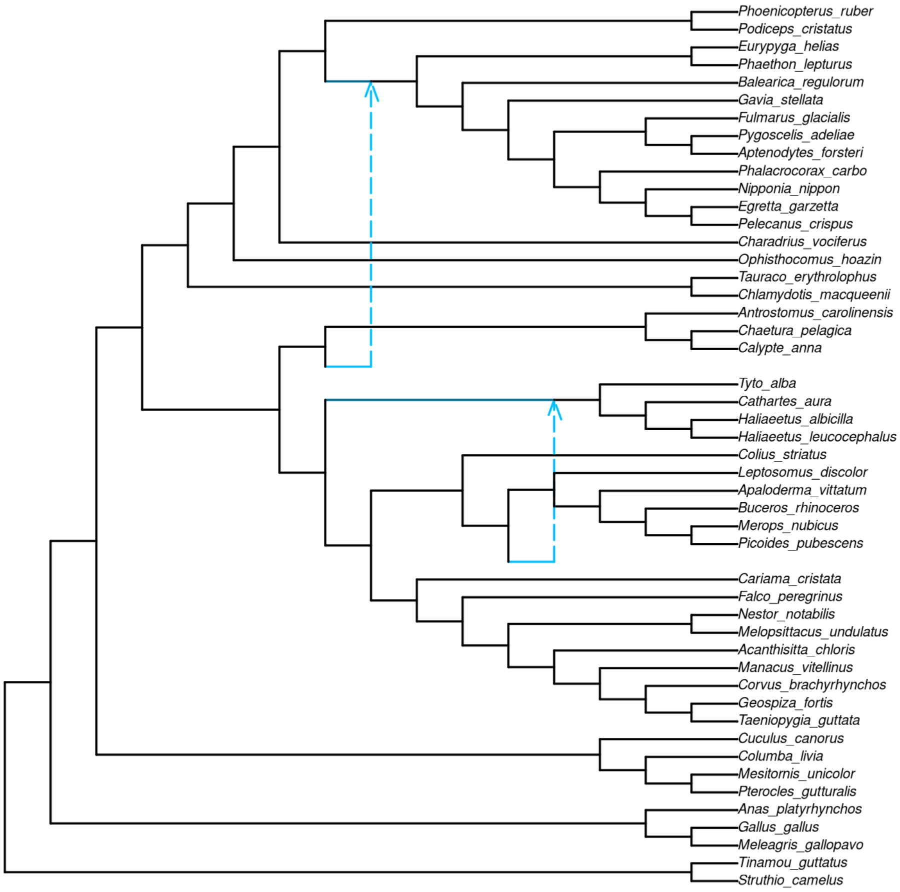

Figure S7: Network returned by CAMUS with ASTRAL constraint tree; $t = 0.8$; edges in gene trees with support less than 75% are collapsed.

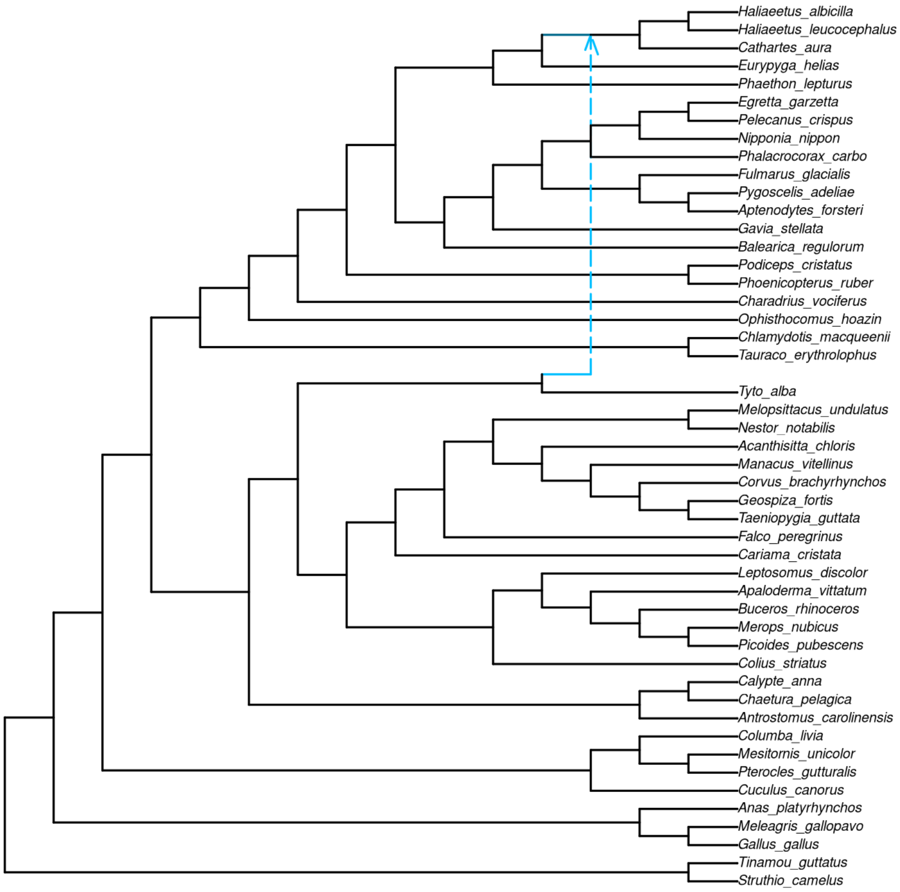

Figure S8: Network with one reticulation returned by PhyloNet-MPL (FT) with ASTRAL constraint tree; 1 reticulation maximum; edges in gene trees with support less than 75% are collapsed.

4.3 PhyloNet-MPL(FT) analyses of the Avian dataset

In this section, we show PhyloNet-MPL(FT) analyses of the Avian dataset, given the rooted ASTRAL species tree and using gene trees where all edges of support less than 75% are collapsed.

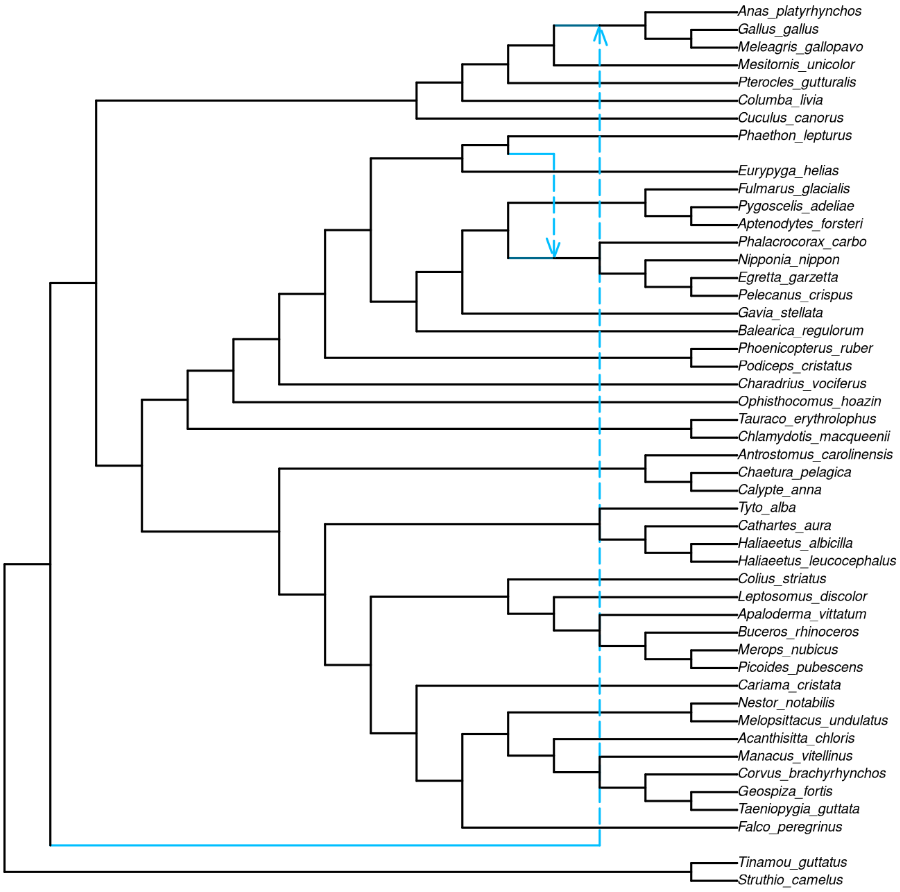

Figure S9: Network returned by PhyloNet-MPL (FT) with ASTRAL constraint tree; 2 reticulation maximum; edges in gene trees with support less than 75% are collapsed.

5 References

[1] Joshua A Justison, Claudia Solis-Lemus, and Tracy A Heath. SiPhyNetwork: An R package for simulating phylogenetic networks. *Methods in Ecology and Evolution*, 14(7):1687–1698, 2023.

[2] John Fogg, Elizabeth S Allman, and Cécile Ané. PhyloCoalSimulations: a simulator for network multispecies coalescent models, including a new extension for the inheritance of gene flow. *Syst Biol*, 72(5):1171–1179, 2023.

[3] William Fletcher and Ziheng Yang. INDELible: a flexible simulator of biological sequence evolution. *Molecular biology and evolution*, 26(8):1879–1888, 2009.

[4] Morgan N Price, Paramvir S Dehal, and Adam P Arkin. FastTree 2–approximately maximum-likelihood trees for large alignments. *PloS one*, 5(3):e9490, 2010.

[5] Thomas KF Wong et al. IQ-TREE 3: Phylogenomic inference software using complex evolutionary models, 2025. Published on EcoEvoRxiv, `https://doi.org/10.32942/X2P62N`.

[6] James Willson, Mrinmoy Saha Roddur, Baqiao Liu, Paul Zaharias, and Tandy Warnow. DISCO: species tree inference using multicopy gene family tree decomposition. *Systematic Biology*, 71(3):610–629, 2022.

[7] Simon Tavaré. Some probabilistic and statistical problems in the analysis of DNA sequences. *Lectures on Mathematics in the Life Sciences*, 17:57–86, 1986.

[8] Chao Zhang, Rasmus Nielsen, and Siavash Mirarab. ASTER: a package for large-scale phylogenomic reconstructions. *Molecular Biology and Evolution*, 42(8):msaf172, 2025.

[9] Niema Moshiri. TreeSwift: A massively scalable Python tree package. *SoftwareX*, 11:100436, 2020.

[10] Claudia Solís-Lemus and Cécile Ané. Inferring phylogenetic networks with maximum pseudolikelihood under incomplete lineage sorting. *PLoS genetics*, 12(3):e1005896, 2016.

[11] Claudia Solís-Lemus, Paul Bastide, and Cécile Ané. Phylonetworks: a package for phylogenetic networks. *Molecular biology and evolution*, 34(12):3292–3298, 2017.

[12] Cuong Than, Derek Ruths, and Luay Nakhleh. PhyloNet: a software package for analyzing and reconstructing reticulate evolutionary relationships. *BMC bioinf*, 9(1):322, 2008.

[13] Yun Yu and Luay Nakhleh. A maximum pseudo-likelihood approach for phylogenetic networks. *BMC genomics*, 16:1–10, 2015.

[14] Erich D Jarvis et al. Whole-genome analyses resolve early branches in the tree of life of modern birds. *Science*, 346(6215):1320–1331, 2014.

[15] Erich D Jarvis et al. Phylogenomic analyses data of the avian phylogenomics project. *GigaScience*, 4(1):s13742–014, 2015.
